# Are contouring time and multimodality imaging prognostic factors for radiation therapy of advanced head and neck cancer?

**DOI:** 10.1186/1470-7330-15-S1-P28

**Published:** 2015-10-02

**Authors:** Y Eller, ND Klass, M Schmuecking, O Elicin, R Bigler, J Tille, S Fankhauser, N Mertineit, B Klaeser, A Geretschlaeger

**Affiliations:** 1 Department of Radiation Oncology, Inselspital, Bern University Hospital and University of Bern, Bern, Switzerland; 2 Department of Radiology and Radiation Oncology, Radiation Center Hamburg / CyberKnifeCenter Hamburg, Hamburg, Germany; 3 Division of Medical Radiation Physics, Inselspital, Bern University Hospital and University of Bern, Bern, Switzerland; 4 Department of Radiology, Inselspital, Bern University Hospital and University of Bern, Bern, Switzerland; 5 Department of Nuclear Medicine, Inselspital, Bern University Hospital and University of Bern, Bern, Switzerland; 6 Department of Radiation Oncology, St. Claraspital, Basel, Switzerland

## Background

To evaluate if contouring time and multimodality imaging are prognostic factors for radiation therapy of advanced head and neck cancer (HNC) 207 patients were analyzed retrospectively between 2001-2012.

## Material and methods

Before 2007 radiation treatment planning-CT was done without contrast enhancement, MRI and ^18^F-FDG-PET/CT were used only occasionally. From 2007 contrast enhanced planning-CT in addition to multimodality imaging was used routinely for every HNC. Additionally, in unclear or equivocal imaging findings of lymph nodes a re-report was performed with a higher sensitivity at the expense of specificity to minimise geographical miss in the contouring procedure for radiation treatment and to maximise the binary decisions for each lymph node (malignant vs. benign). The re-reports were done in conjunction with radio oncologists, nuclear physicians and radiologists. The mean contouring time was 60min before 2007, 150min after 2007 (including the time for a re-report). Clinical outcome was assessed in two groups (group I: 2001-2007, n=113 vs. group II: 2008-2012, n=94).

## Results

Regional control was significantly higher in group II (log-rank-test p=0.03): group I after 3 years 76%, group II after 3 years 88%. Locoregional control for 207 patients shows no difference in survival (p=0.08); however, inclusion of 340 patients would lead to a p-value p<0.05.

## Conclusions

Imaging findings of multimodality imaging and a critical re-report of these imaging findings in conjunction with a longer contouring time may have an impact on clinical outcome. However, this overtime is not reimbursed. A close collaboration of radiooncologists, nuclear physicians and radiologists in the radiation treatment planning process may have a benefit for patients with advanced HNC.

